# Vitamin A deficiency execrates Lewis lung carcinoma via induction of type 2 innate lymphoid cells and alternatively activates macrophages

**DOI:** 10.1002/fsn3.961

**Published:** 2019-02-10

**Authors:** Weiwei Cui, Wenxin Zhang, Xiaofeng Yuan, Shanshan Liu, Meng Li, Junqi Niu, Peng Zhang, Dong Li

**Affiliations:** ^1^ Department of Nutrition and Food Hygiene, School of Public Health Jilin University Changchun China; ^2^ Department of Pathology The First Hospital of Jilin University Changchun China; ^3^ Department of Pediatrics Affiliated Hospital of Changchun University of Chinese Medicine Changchun China; ^4^ Department of Immunology, College of Basic Medical Sciences Jilin University Changchun China; ^5^ Department of Epidemiology and Biostatistics, School of Public Health Jilin University Changchun China; ^6^ Department of Hepatology The First Hospital of Jilin University Changchun China; ^7^ Department of Thoracic Surgery The First Hospital of Jilin University Changchun China; ^8^ Department of Pathology University of Cambridge Cambridge UK

**Keywords:** alternatively activated macrophages, cytokine, ILC2, lung carcinoma, vitamin A

## Abstract

**Background:**

Lung carcinoma is still associated with high rates of morbidity and mortality despite the advances in cancer therapy achieved in last decades. Recent studies showed that immune responses played a crucial role in the developments of cancers including lung cancer. Type 1 immune response could promote classical activated macrophages (CAMs) with antitumor properties. On the contrast, type 2 immune response could lead to the polarization of alternatively activated macrophages (AAMs) which could promote the growth and metastasis of tumor. Our previous research showed that vitamin A deficiency could promote the type 2 immune response but not the type 1 immune response. Whether vitamin A deficiency has detrimental effect for lung carcinoma need further investigate.

**Aim:**

To investigate the effect of vitamin A deficiency in lung cancer and the potential mechanisms.

**Methods:**

Mice were fed with normal diet or vitamin A deficiency diet for 2 weeks, and then, Lewis lung cancer (LLC) cells dissolved in Matrigel Matrix were planted on the left lower lope of lungs. Mice were sacrificed 28 days after the plantation of tumor cells, the tumor size, cytokine profile in bronchoalveolar lavage fluid (BALF), numbers of type 2 innate lymphoid cells (ILC2s), and macrophage phenotypes in the lung were measured. The overall survival rate was also monitored throughout the experiments.

**Results:**

Vitamin A deficiency diet fed tumor‐bearing mice have lower survival rate (*χ* ^2^ = 6.862, *p* < 0.001), larger tumor size (*t* = 2.651, *p* < 0.05), more ILC2s (*t* = 7.680, *p* < 0.001), and AAMs (*t* = 6.315, *p* < 0.001) in the lung tissue; also, type 2 cytokines concentrations in the BALF were higher compared to normal diet fed ones.

**Conclusion:**

Vitamin A deficiency could promote the pathogeneses of lung carcinoma via induction of ILC2s and polarizing AAMs.

## 1 INTRODUCTION

Lung carcinoma is still one of the leading causes of deaths in China, despite the advances made in recent decades (Chen et al., 2016). The exact etiology of lung carcinoma is still not fully understood yet, but it was generally believed to be associated with both genetic and environmental factors (Cheng et al., 2012). Recent studies about the interactions of immune system and tumors, including lung cancer, showed that the fate of tumors or even the host would be determined by immune system that infiltrated and surrounded tumors (Joyce & Fearon, 2015; Kitamura, Qian, & Pollard, 2015; Lim & June, 2017). The immune cells could either eliminate the tumors or help the tumor growth and metastasis which might even lead to the death of the host. The resurrection of tumor immune therapy in recent decade proved that it is possible to treat tumors via manipulating the immune system (Bender, 2017; Couzin‐Frankel, 2013). Although lots of current studies focused on the cytotoxic T lymphocytes, like the work did on the blockage of PD‐1/PDL‐1 and CTLA‐4 (Pardoll, 2012), there are other immune cells that could regulate the immune system and even kill the tumor cells directly, like the most essential immune cell, macrophages (Mantovani, Marchesi, Malesci, Laghi, & Allavena, 2017).

Macrophages play important roles during all stages of immune responses; it could initiate, maintain, or terminate the immune responses (Wynn, Chawla, & Pollard, 2013). Traditionally, the macrophages could be cataloged to two distinct subsets, one is the pro‐inflammatory M1 or classically activate macrophages (CAM) which could be polarized by type I cytokines such as IL‐12 and INFγ; another one is anti‐inflammatory, pro‐resolution, and pro‐fibrotic M2 or alternately activate macrophages (AAM) which could be polarized by type II cytokines such as IL‐4, IL‐5, and IL‐13 (Wynn et al., 2013; Wynn & Vannella, 2016). Both CAMs and AAMs are indispensable for maintaining the balance of immune system. The immune system needs the CAMs for their anti‐infection or antitumor properties for the protection and the AAMs to limit the immune responses to avoid the damage to self‐cell (Hume, 2015; Vannella & Wynn, 2017). Macrophages also could play the determination role in shaping the immune response during the pathogenesis of tumors. They could have either detrimental or beneficial effects depend on the specific polarization conditions (Murray, 2017). CAMs were reported to be able to suppress the growth of tumors, yet the AAMs were reported to be able to promote the growth and metastasis of tumors (Mantovani et al., 2017).

Diet might contribute to the education and regulation of immune system (Julia, Macia, & Dombrowicz, 2015; Pan et al., 2017). Vitamin A (VA) and its metabolic product retinoic acid (RA) were reported to be the essential nutrients for the development and maturation of variety types of immune cells (Canete, Cano, Munoz‐Chapuli, & Carmona, 2017). Previous studies also showed that dietary vitamin A intake could indeed reduce lung cancer risk, but the underlining mechanisms were still largely unknown (Yu, Su, Wang, Dai, & Kang, 2015). Our previous study showed that deficient in VA could lead to increase an enhanced type II immune response during allergic diseases (Cui et al., 2016). And type II immune responses might link to the polarization of AAMs which could promote the tumor cells growth and metastasis (Brune, Weigert, & Dehne, 2015; Kurowska‐Stolarska et al., 2009; Li, Guabiraba, et al., 2014; Mantovani et al., 2017).

In this work, we established Lewis lung carcinoma model via planting LLC cells directly on the left lower lope of lungs of C57/B6 mice. And we compared the tumor growth and survival rate mice fed with vitamin A deficiency (VAD) diet or control diet received LLC cells or matrix gel controls. We also measure the BALF cytokine profiles, type 2 innate lymphoid cells (ILC2s), and macrophages of these mice to investigate the role of vitamin A in lung carcinoma.

## 2 MATERIAL AND METHODS

### 2.1 Mice

Specific‐pathogen‐free male C56BL/6 mice weighing 20–22 g (purchased from Beijing Vital River Laboratory Animal Technology Co., Ltd., Beijing, China) were housed in specific‐pathogen‐free conditions at Jilin University, China. All experimental were performed in accordance with the National Guidelines for Experimental Animal Welfare and with approval of the Animal Welfare and Research Ethics Committee at Jilin University (Cui et al., 2016; Li et al., 2016).

Vitamin A‐sufficient (TD.10992) and Vitamin A‐deficient (TD.10991) diets were purchased from Harlan Laboratories, Inc., WI, USA (Cui et al., 2016; Spencer et al., 2014). All mice were maintained on special diet and autoclaved distilled water from the day of arrival until the end of experiments, and all other procedures were started at least 14 days afterward, respectively.

### 2.2 Enzyme‐linked immunosorbent assay

The concentrations of cytokines in BAL fluid were determined by enzyme‐linked immunosorbent assay (ELISA, eBioscience, Inc., CA, USA) according to manufacturers’ instructions as described previously (Cui et al., 2016; Li et al., 2016; Li, Guabiraba, et al., 2014; Pushparaj et al., 2013).

### 2.3 Lewis lung carcinoma model

The Lewis lung cancer model was established as described (Bobek et al., 2010). Briefly, LLC cells were cultured in complete media (DMEM with 10% heat‐inactivated FBS, 100 U/ml Penicillin, 100 μg/ml Streptomycin, 2 mM L‐Glutamine; Sigma‐Aldrich, MO, USA). Cultures were incubated at 37.0°C in a humidified incubator (Thremo Fishher, Germany) supplemented with 5% CO_2_. Viable cells were counted with a Neubauer haemocytometer on a Nikon Labphot microscope, staining with 0.1% (w/v) trypan blue (Sigma‐Aldrich, MO, USA).

Mice were anesthetized by intraperitoneal (i.p.) injections of 150 μl of Avertin (20 mg/ml 2,2,2‐Tribromoethanol, Sigma‐Aldrich, MO, USA). Then the lungs were exposed by surgically opened around 0.5‐cm longitude cut at left chest, 6 × 10^5^ LLC cells suspended in 20 μl of BD Matrigel (Becton Dickinson, NJ, USA), or Matrigel alone (for control groups) were injected on the left lower lope of lungs. The surgical wounds were stitched up with a suture. And the mice were closely monitored until fully recovered from anesthesia. Mice were sacrificed 28 days after the injection of LLC cells by i.p. injection of 500 μl of Avertin. Bronchoalveolar lavage (BAL) fluid and lung tissues were collected and analyzed as previously described (Cui et al., 2016; Li, Guabiraba, et al., 2014). The tumor size was calculated by using the following formula, *V* = 0.5 × *L* × *W* ^2^, where *L* and *W* are the length and width of tumor (Li, Tian, et al., 2014).

### 2.4 Quantitative PCR

RNA was purified from tissue samples using the RNeasy Mini Kit following the manufacturer's instructions as described previously (Qiagen, UK; Cui et al., 2016; Li, Guabiraba, et al., 2014). Reverse transcription (RT) of RNA into cDNA was carried out using High‐Capacity cDNA Reverse Transcription Kits (Thermo Fisher Scientific, MA, USA). Real‐time polymerase chain reaction (RT‐PCR) was performed using Fast SYBR Green master mix on a Prism 7900HT (Thermo Fisher Scientific). The primers used were as follows: *Il4*, forward 5′‐CAT GGC TTG GGT ACA GGT CT‐3′, reverse 5′‐TTT GTA GTG GGA GGG GAC AG‐3′; *Il5*, forward 5′‐GAA GTG TGG CGA GGA GAG AC‐3′, reverse 5′‐GCA CAG TTT TGT GGG GTT TT‐3′; *Il13*, forward 5′‐GAA TCC AGG GCT ACA CAG AAC‐3′, reverse 5′‐AAC ATC ACA CAA GAC CAG ACT C‐3′; *Ifng*, forward 5′‐ACT TTG CTT CTG CCT TTC CA‐3′, reverse 5′‐ACA AGG TCA CCC ACA GGA‐3′.

### 2.5 Computed tomographic scanning

Mice were kept in pathogen‐free conditions for 21 days after the LLCs were administrated; the development of lung cancer was determined by using computed tomographic (CT) scans. Scans were performed with a CT scanner (SOMATOM Definition AS, Siemens Healthcare GmbH, Germany), as previously described. Mice were anesthetized and placed in the prone position on the micro‐CT bed without respiratory gating. The tube voltage was 120 kV, current was 35 mA, and exposure time was 5 s.

### 2.6 Flow cytometry

The leukocytes infiltrated to the lungs were analyzed by flow cytometry as previously described (Li, Guabiraba, et al., 2014). Briefly, lungs were harvested on day 14 after LLCs administration and digested in 125 mg/ml Liberase TL and 100 mg/ml DNAse 1 (Roche Diagnostics, Switzerland) to characterize the infiltrating leukocytes. Dispersed cells (1 × 10^6^ cells per tube) were stained with fluorochrome‐conjugated mAbs against CD45‐PerCP, CD11b‐APC (all antibodies used in flow cytometry were purchased from BD, NJ, USA. unless otherwise indicated), F4/80‐FITC (eBioscience, CA, USA), and CD206‐PE (BioLegend, CA, USA). Leukocytes were stained with antibodies against ST2–FITC (MD Biosciences, MN, USA), lineage markers (CD3, B220, CD11b, CD11c, FcεR1, SIGLEC‐F) labeled with PE, CD45‐APC, and ICOS‐PerCP (BioLegend) to characterize the infiltrating ILC2s. Cells were analyzed with a BD carlibr Analyzer (BD). Gating strategy (Supporting Information Figure S1) and analysis were performed with FlowJo software (FlowJo LLC OR, USA).

### 2.7 Statistical analysis

Analysis between in vivo groups was examined by Mann–Whitney *U* test or ANOVA followed by Student's *t* test using GraphPad Prism software (Cui et al., 2016; Li et al., 2016; Li, Guabiraba, et al., 2014). Survival analysis *p* values were calculated using the log‐rank (Mantel–Cox) test. All data are expressed as means ± SEM. Values of *p* < 0.05 were considered significant.

## 3 RESULTS

### 3.1 Vitamin A deficiency exacerbates the tumor cell growth

**Figure 1:**
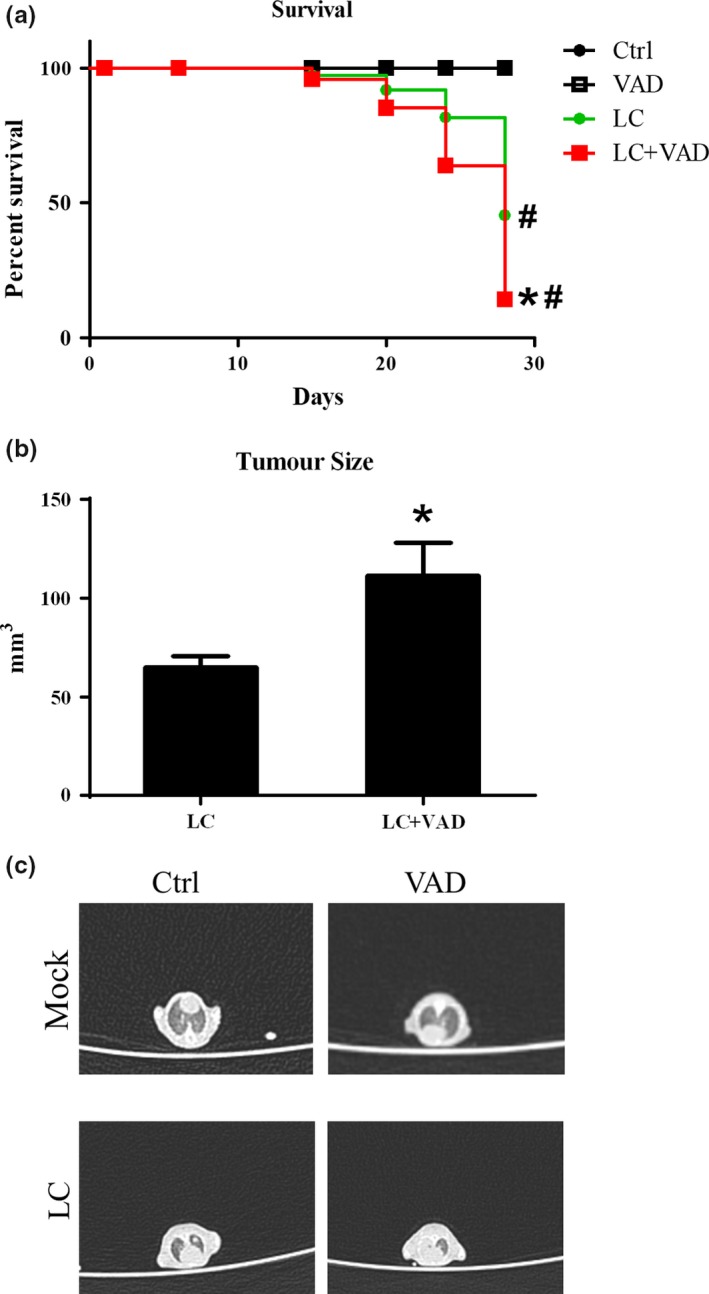
Vitamin A deficiency enhanced the progress of Lewis lung cancer. After the mice were fed with VAD or control diet for 14 days, the LLC cells (LC) or control matrix gel (Mock) were injected into the lung; (a) the survival rates of the mice were monitored; (b) the sizes and of tumors in the left lower lope of lungs were measured at 28 days after the LLC administrations; and (c) the tumor formation was also confirmed by the CT scan. Vertical bars = SEM, *n* = 10–12 mice/group/experiment, *p < 0.05 compared to normal diet fed group(s); #*p *< 0.05 compared to control matrix gel group(s). Data are representative of three experiments

Our previous study showed that vitamin A deficiency (VAD) could enhance the type II immune response and exacerbate the diseases progress in an asthma mice model (Cui et al., 2016). To investigate if the similar phenomena could be observed in tumor, we fed the mice with VAD or control diet for two weeks before the administration of tumor cells. The mice in VAD group had a significant lower survival rate compared to normal diet group after received tumor cells (*χ* ^2^ = 6.862, *p* < 0.001), and the mice only received matrix gel did not die during the length of experiment (Figure 1a). And the tumor size was significantly larger in VAD group as well (*t* = 2.651, *p* < 0.05; Figure 1b,c).

### 3.2 Vitamin A deficiency enhanced the immune cells infiltration to the lung

**Figure 2:**
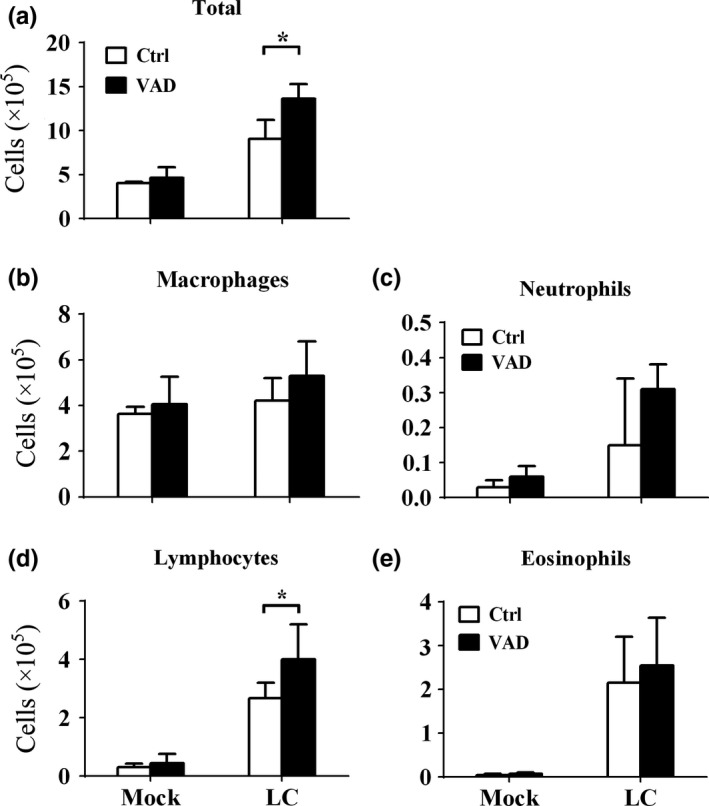
Vitamin A deficiency affected the leukocytes infiltrations to the lung during Lewis lung cancer. After the mice were fed with VAD or control diet for 14 days, the LLC cells (LC) or control matrix gel (Mock) were injected into the lung, and the mice were terminated 28 days later. The BALF was collected, and the cells in BALF were analyzed. And the (a) total cell counts; numbers of (b) macrophages, (c) neutrophils, (d) lymphocytes, and (e) eosinophils in each group were shown. Vertical bars = SEM, *n* = 10–12 mice/group/experiment, *p < 0.05 compared to normal diet fed group. Data are representative of three experiments

After established that feeding with VAD diet could exacerbate the lung cancer in mice. We next investigate how the VAD could influent the recruitment of immune cells in the lung. The BALF was harvested after the mice were terminated, and the cells in BALF were analyzed. The total cell number was increased by VAD (*t* = 13.43, *p* < 0.001; Figure 2a). As for each cell types, only lymphocytes number was increased significantly in VAD groups of tumors‐bearing mice (*t* = 4.775, *p* < 0.001; Figure 2d); the number of macrophages, neutrophils, and eosinophils were increased slightly by VAD but not statistically significant (*t* = 2.081, *p* > 0.05; *t* = 2.190, *p* > 0.05; *t* = 1.236, *p* > 0.05; Figure 2b,c,e).

### 3.3 Vitamin A deficiency enhanced the type II cytokine levels in the lung

**Figure 3:**
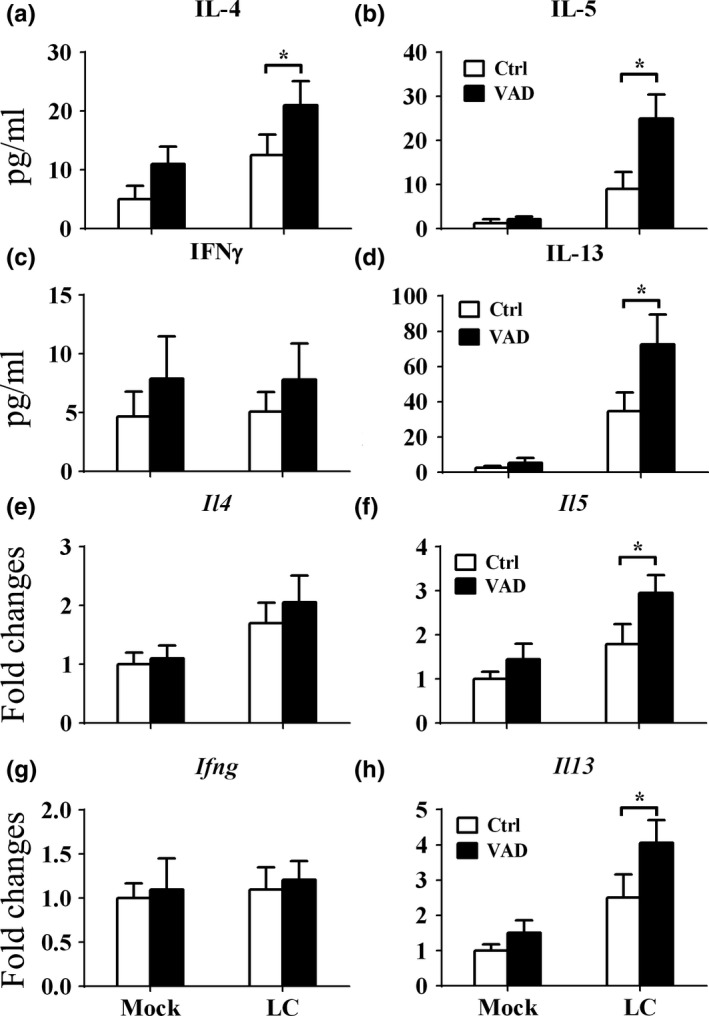
Vitamin A deficiency enhanced the production of type 2 cytokines in the lungs in Lewis lung cancer. After the mice were fed with VAD or control diet for 14 days, the LLC cells (LC) or control matrix gel (Mock) were injected into the lung, and the mice were terminated 28 days later. The BALF and left lower lope of lungs were collected, and then, the cytokine concentrations (a–d) in the BALF and mRNA gene expressions (e–h) of these cytokines in the lung tissue were both analyzed. Vertical bars = SEM, *n* = 10–12 mice/group/experiment, *p < 0.05 compared to normal diet fed group. Data are representative of three experiments

After established that VAD exacerbates tumor growth and immune cells infiltrations to the lung. We next check the cytokines profiles in the BALF, only type II cytokines such as IL‐4, IL‐5, and IL‐13 were up‐regulated by VAD (*t* = 2.329, *p* < 0.05; *t* = 3.501, *p* < 0.01; *t* = 2.788, *p* < 0.05; Figure 3a,b,d), but not type I cytokine such as IFNγ (*t* = 0.6543, *p* > 0.05; Figure 3c). The gene expressions of these cytokines in lung tissue were also analyzed. Very similar to protein analyses, the expressions of *Il4*, *Il5,* and *Il13* (*t* = 2.543, *p* < 0.05; *t* = 2.608, *p* < 0.05; *t* = 2.445, *p* < 0.05) were increased but not *Ifng* (*t* = 0.2860, *p* > 0.05; Figure 3e–h).

### 3.4 Vitamin A deficiency enhanced the ILC2s and AAMs infiltration to the lung

**Figure 4:**
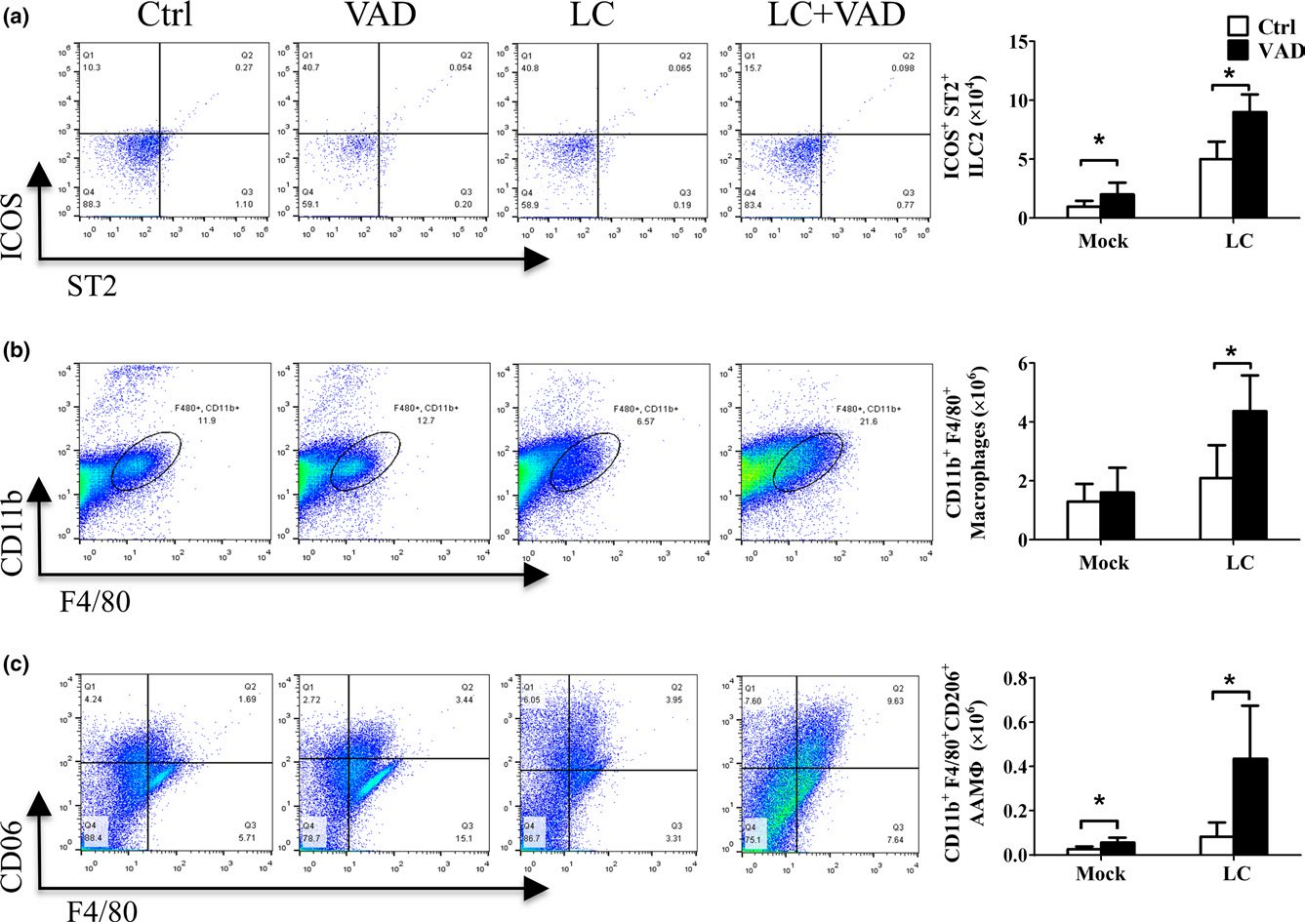
Vitamin A deficiency induced the ILC2s and AAM infiltrations to the lungs in Lewis lung cancer. After the mice were fed with VAD or control diet for 14 days, the LLC cells (LC) or control matrix gel (Mock) were injected into the lung, and the mice were terminated 14 days later. The lower lope of lungs was collected, and the leukocytes were analyzed by flow cytometry. Representative figures from the flow cytometry analysis and cell numbers were shown for (a) ILC2s, (b) macrophages, and (c) alternatively activated macrophages. Vertical bars = SEM, *n* = 10–12 mice/group/experiment, *p < 0.05 compared to normal diet fed group. Data are representative of three experiments

Next, the cells in lung tissue were analyzed by flow cytometry to investigate how VAD affect the lung cancer. ILC2s were recruited to the lung by VAD (*t* = 7.680, *p* < 0.001; Figure 4a) as reported before in intestine (Spencer et al., 2014). The macrophages numbers in lung were also enhanced by VAD (*t* = 5.294, *p* < 0.001; Figure 4b). And the up‐regulated type II cytokines productions in the lung by VAD was also associated with increased AAM cell infiltrations to the lung (*t* = 6.315, *p* < 0.001; Figure 4c).

## 4 DISCUSSIONS

In this study, we found that VAD could increase ILC2s infiltrations to the lung, which might be related to the enhanced type II cytokines productions in the lung, in turn might increase alternately activated macrophages in the lung, eventually leading to the exacerbation of tumor growth and decreased survival rate of tumors‐bearing mice.

Vitamin A deficiency is one of the most common malnutrition around the world, which proposes a major thread in public health (Hall, Grainger, Spencer, & Belkaid, 2011). Vitamin A and its metabolite RA are required for the immune system to perform its functions during both physiological and pathological conditions (Canete et al., 2017; Gundra et al., 2017). Especially for the adaptive immune system, T cells need RA for their maturation, recruitment, and polarization (Iwata, Mukai, Nakai, & Iseki, 1992). But individuals suffered from vitamin A deficiency still could live for a considerably long time, indicating that the immune system has some mechanisms to compensate the loss of functions from vitamin A deficiency (Canete et al., 2017). Recent studies found that the helper innate lymphoid cells could replace the T cells during infections to maintain the protection role of immune systems (Artis & Spits, 2015). And it is the ILC2s, not ILC1 or ILC3 that selectively proliferated during vitamin A deficiency conditions (Spencer et al., 2014). The reason for this may be the function of ILC2s is to obstruct a type 2 immune response with or without the T cells, and it is crucial for the protection against the helminths infections (Klose & Artis, 2016; Wynn, 2015).

The over‐activated ILC2s during noninfectious conditions could have some detrimental effects (Artis & Spits, 2015). It could exacerbate or even initiate the diseases such as asthma or fibrosis (Li, Guabiraba, et al., 2014). Our previous works showed that during asthmatic diseases, the vitamin A deficiency enhanced the type 2 immune responses which might partially due to the proliferation and activation of ILC2s (Cui et al., 2016). And during both liver and lung fibrosis, ILC2s polarized the alternate activated macrophages (AAMs) and together they exacerbate the fibrotic diseases (Li, Guabiraba, et al., 2014; McHedlidze et al., 2013). So, in this work we tried to investigate whether vitamin A deficiency could lead to the proliferation and activation of ILC2s and AAMs during the pathogenesis of tumors.

It has been well established that the interaction between tumor cells and immune cells could determine the final fate of tumor (Mantovani et al., 2017). It could be eliminated by immune cells or helped by immune cells to grow uncontrollably or metastasis to other organs (Lim & June, 2017). Although a lot of factors get involved in these processes, the macrophages, which were known to have pleiotropic functions, might play the dominant role here (Wynn & Vannella, 2016). The tumor infiltrating or surrounding macrophages could kill tumor cells directly or indirectly via recruiting, activating, and polarizing other immune cells, or they could help the tumor cells escape from other immune cells, depending which subsets the macrophages have been polarized into (Brune et al., 2015). Further understanding of how macrophages were polarized could help to alter the polarization status of macrophages and help in the treatment of tumors.

To sum up, this work provided more insight into how nutrients such as vitamin A and RA reshaped the immune responses. These findings may provide novel strategy for the treatments of lung carcinoma.

## CONFLICTS OF INTEREST

The authors declare that they do not have any conflict of interest.

## ETHICAL REVIEW

This study was performed in accordance with the National Guidelines for Experimental Animal Welfare and with approval of the Animal Welfare and Research Ethics Committee at Jilin University.
