# Supplementary figures and images for: Vitamin A deficiency execrates Lewis lung carcinoma via induction of type 2 innate lymphoid cells and alternatively activates macrophages
Source: Food Sci Nutr. 2019 Feb 10;7(4):1288–94. doi: 10.1002/fsn3.961 (PMC6475724; doi:10.1002/fsn3.961)

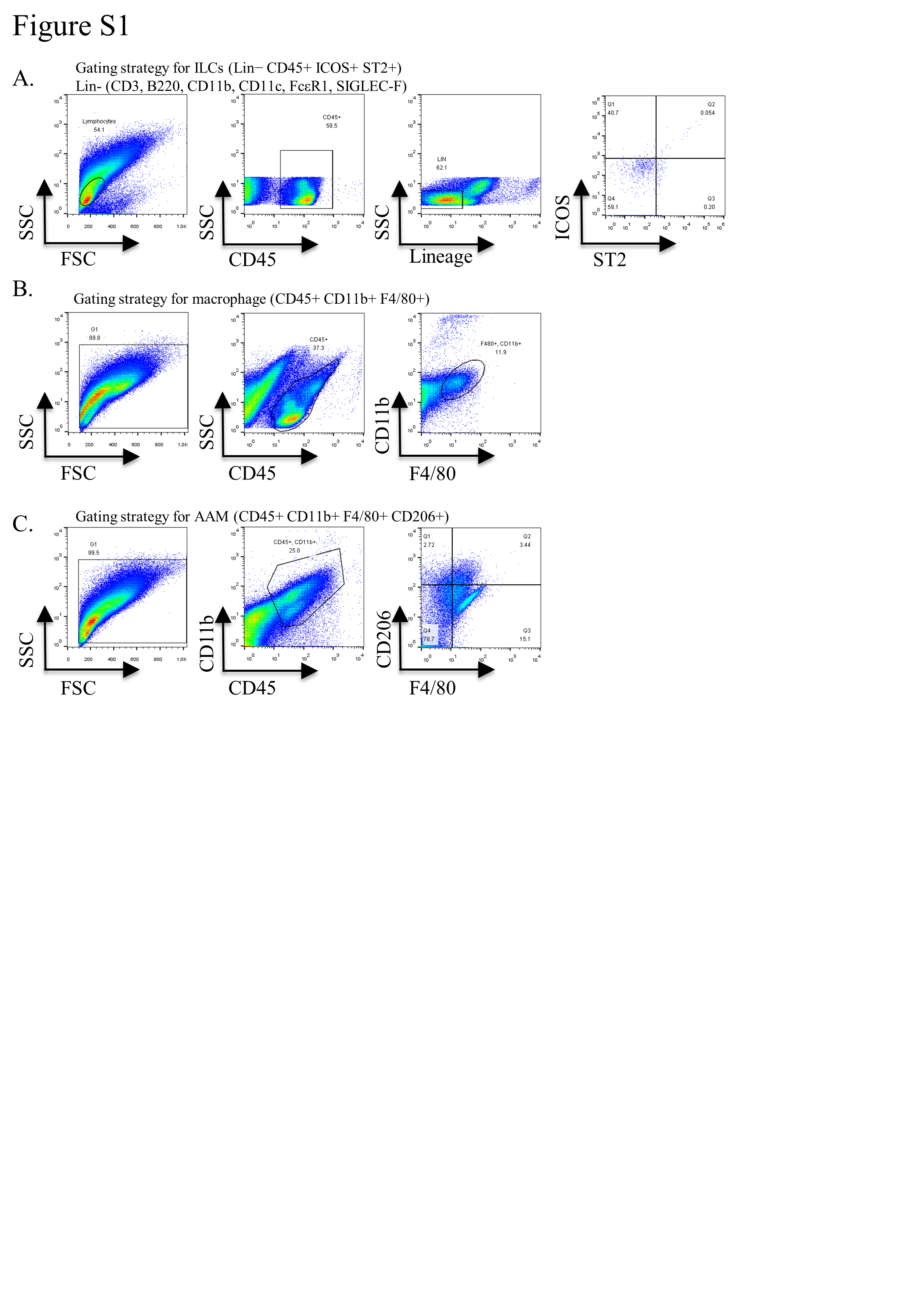

Supplement: Supplementary file 1 [file FSN3-7-1288-s001.tif]
